# Supplementary material for: Excessive G–U transversions in novel allele variants in SARS-CoV-2 genomes
Source: PeerJ. 2020 Jul 28;8:e9648. doi: 10.7717/peerj.9648 (PMC7394058; doi:10.7717/peerj.9648)

Table S1. List of accessions used in multiple alignments.

| SARS-CoV-2 genomes | SARS-CoV genomes | HKU1 genomes |
| --- | --- | --- |
| MT121215.1 | AY395003.1 | KY674921.1 |
| MT334529.1 | AY394996.1 | MH940245.1 |
| MT385430.1 | AY304488.1 | DQ415911.1 |
| MT371050.1 | AY304486.1 | AY884001.1 |
| MT135041.1 | AY390556.1 | DQ415913.1 |
| MT385425.1 | EU371564.1 | DQ415902.1 |
| MT114413.1 | AY394985.1 | DQ339101.1 |
| MT192772.1 | AY278554.2 | MK167038.1 |
| MT135043.1 | EU371559.1 | DQ415912.1 |
| MT049951.1 | AY559093.1 | DQ415903.1 |
| MT385419.1 | AY394994.1 | DQ415899.1 |
| MT385421.1 | AY394986.1 | DQ415898.1 |
| MT114412.1 | JX163927.1 | DQ415897.1 |
| MT385424.1 | JX163926.1 | HM034837.1 |
| MT371049.1 | JX163923.1 | DQ415906.1 |
| MT114418.1 | JQ316196.1 | AY597011.2 |
| MT350282.1 | FJ882963.1 | DQ415908.1 |
| MT339041.1 | DQ898174.1 | DQ415904.1 |
| MT324062.1 | AY864806.1 | KY674943.1 |
| MT281577.1 | AY559096.1 | KY674942.1 |
| MT163719.1 | AY559095.1 | KY674941.1 |
| MT246480.1 | AY559086.1 | KF686346.1 |
| MT334533.1 | AY559085.1 | KF686343.1 |
| MT385423.1 | AY559084.1 | KF686341.1 |
| MT385417.1 | AY559083.1 | DQ415914.1 |
| MT385415.1 | AY559082.1 | DQ415910.1 |
| MT385414.1 | AY323977.2 | DQ415909.1 |
| MT374111.1 | AY291451.1 | DQ415907.1 |
| MT374110.1 | AY502928.1 | DQ415905.1 |
| MT374104.1 | AY502926.1 | DQ415901.1 |
| MT371048.1 | AY502923.1 | DQ415900.1 |
| MT370518.1 | AY394999.1 | DQ415896.1 |
| MT114417.1 | AY394998.1 | KT779556.1 |
| MT334542.1 | AY394995.1 | KT779555.1 |
| MT322401.1 | AY394993.1 | KF686344.1 |
| MT385420.1 | AY394992.1 | KF686342.1 |
| MT334541.1 | AY394991.1 | KF430201.1 |
| MT263396.1 | AY394987.1 | KF686340.1 |
| MT251978.1 | AY394983.1 |  |
| MT246460.1 | AY394978.1 |  |
| MT192773.1 | AY357075.1 |  |
| MT385429.1 | AY282752.2 |  |
| MT385422.1 | AY427439.1 |  |
| MT374115.1 | AY283796.1 |  |
| MT374102.1 | AP006561.1 |  |
| MT358675.1 | JX163925.1 |  |
| MT326187.1 | JX163928.1 |  |
| MT322424.1 | AY559081.1 |  |
| MT322417.1 | JX163924.1 |  |
| MT322405.1 | GU553363.1 |  |
| MT163718.1 | EU371563.1 |  |
| MT385436.1 | EU371561.1 |  |
| MT365033.1 | EU371560.1 |  |
| MT186683.1 | AY864805.1 |  |
| MT370516.1 | AY278741.1 |  |
| MT358637.1 | AY559087.1 |  |
| MT114415.1 | AY502932.1 |  |
| MT114414.1 | AY502930.1 |  |
| MT334544.1 | AY502929.1 |  |
| MT334538.1 | AY395002.1 |  |
| MT328035.1 | AY395000.1 |  |
| MT276324.1 | AY394990.1 |  |
| MT246462.1 | AY394989.1 |  |
| MT385426.1 | AY345986.1 |  |
| MT339039.1 | AY357076.1 |  |
| MT304483.1 | AY350750.1 |  |
| MT374116.1 | AY362699.1 |  |
| MT374114.1 | AY283794.1 |  |
| MT374108.1 | AY283795.1 |  |
| MT334534.1 | AY321118.1 |  |
| MT252780.1 | AY394850.2 |  |
| MT358705.1 | AP006560.1 |  |
| MT334547.1 | AP006559.1 |  |
| MT328032.1 | AP006557.1 |  |
| MT385427.1 | AY485278.1 |  |
| MT385418.1 | AY502927.1 |  |
| MT374113.1 | GU553365.1 |  |
| MT359865.1 | AY502931.1 |  |
| MT322413.1 | AY283797.1 |  |
| MT258381.1 | AY515512.1 |  |
| MT252702.1 | AY394979.1 |  |
| MT374112.1 | AY345988.1 |  |
| MT358715.1 | AY304495.1 |  |
| MT334540.1 | AY283798.2 |  |
| MT326178.1 | AP006558.1 |  |
| MT385416.1 | AY348314.1 |  |
| MT375481.1 | AY338174.1 |  |
| MT375447.1 | AY559092.1 |  |
| MT371047.1 | AY559091.1 |  |
| MT325634.1 | AY559088.1 |  |
| MT325593.1 | MK062179.1 |  |
| MT325592.1 | JN854286.1 |  |
| MT326190.1 | EU371562.1 |  |
| MT304487.1 | AY291315.1 |  |
| MT276328.1 | AY310120.1 |  |
| MT163717.1 | AY278491.2 |  |
| MT123290.1 | AY686864.1 |  |
| MT322409.1 | AY278488.2 |  |
| MT259278.1 | AY338175.1 |  |
| MT259254.1 | AY485277.1 |  |
| MT246466.1 | AY654624.1 |  |
| MT385432.1 | AY545914.1 |  |
| MT385428.1 | DQ497008.1 |  |
| MT374109.1 | AY502924.1 |  |
| MT358702.1 | AY595412.1 |  |
| MT263381.1 | MK062180.1 |  |
| MT385434.1 | DQ640652.1 |  |
| MT252797.1 | AY572035.1 |  |
| MT230904.1 | AY545919.1 |  |
| MT325621.1 | AY545916.1 |  |
| MT325620.1 | AY559094.1 |  |
| MT325619.1 | MK062181.1 |  |
| MT325617.1 | AY461660.1 |  |
| MT326117.1 | AY686863.1 |  |
| MT263395.1 | AY572034.1 |  |
| MT258380.1 | AY545917.1 |  |
| MT246452.1 | MK062183.1 |  |
| MT123291.2 | FJ959407.1 |  |
| MT375474.1 | DQ182595.1 |  |
| MT374107.1 | AY572038.1 |  |
| MT263429.1 | AY545918.1 |  |
| MT258377.1 | AY559090.1 |  |
| LC534418.1 | FJ882938.1 |  |
| MT385433.1 | MK062182.1 |  |
| MT252748.1 | AY279354.2 |  |
| MT345819.1 | AY278490.3 |  |
| MT322419.1 | MK062184.1 |  |
| MT276331.1 | FJ882957.1 |  |
| MT258378.1 | FJ882949.1 |  |
| MT246478.1 | FJ882947.1 |  |
| MT385438.1 | FJ882936.1 |  |
| MT380731.1 | FJ882935.1 |  |
| MT252779.1 | FJ882934.1 |  |
| MT358736.1 | FJ882933.1 |  |
| MT350239.1 | FJ882927.1 |  |
| MT350280.1 | AB257344.1 |  |
| MT350279.1 | AY278487.3 |  |
| MT350274.1 | AY613950.1 |  |
| MT344946.1 | AY613948.1 |  |
| MT325640.1 | FJ882961.1 |  |
| MT325618.1 | FJ882952.1 |  |
| MT325615.1 | FJ882948.1 |  |
| MT325614.1 | FJ882939.1 |  |
| MT325609.1 | FJ882937.1 |  |
| MT325591.1 | FJ882932.1 |  |
| MT325576.1 | AY568539.1 |  |
| MT325566.1 | JF292915.1 |  |
| MT304476.1 | JF292909.1 |  |
| MT304475.1 | FJ882958.1 |  |
| MT304474.1 | AY559089.1 |  |
| MT262900.1 | HQ890541.1 |  |
| LC534419.1 | FJ882945.1 |  |
| MT374106.1 | FJ882930.1 |  |
| MT358402.1 | FJ882926.1 |  |
| MT359866.1 | AY278489.2 |  |
| MT358694.1 | FJ882928.1 |  |
| MT345877.1 | AY613949.1 |  |
| MT325579.1 | AY613947.1 |  |
| MT322418.1 | JF292906.1 |  |
| MT322410.1 | JF292903.1 |  |
| MT246467.1 | HQ890538.1 |  |
| MT246454.1 | HQ890535.1 |  |
| MT163716.1 | HQ890532.1 |  |
| MT385435.1 | HQ890531.1 |  |
| MT375446.1 | HQ890529.1 |  |
| MT344961.1 | HQ890526.1 |  |
| MT325613.1 | FJ882940.1 |  |
| MT325563.1 | AY297028.1 |  |
| MT325562.1 | JF292905.1 |  |
| MT322397.1 | FJ882943.1 |  |
| MT093571.1 | AY772062.1 |  |
| MT375453.1 | FJ882953.1 |  |
| MT320538.2 | FJ882951.1 |  |
| MT358740.1 | JF292922.1 |  |
| MT350278.1 | FJ882962.1 |  |
| MT350277.1 | FJ882942.1 |  |
| MT350276.1 | FJ882931.1 |  |
| MT350273.1 | AY313906.1 |  |
| MT350272.1 | FJ882956.1 |  |
| MT350270.1 | FJ882955.1 |  |
| MT350267.1 | FJ882960.1 |  |
| MT350266.1 | FJ882950.1 |  |
| MT350265.1 | FJ882959.1 |  |
| MT350264.1 | FJ882944.1 |  |
| MT350263.1 | FJ882941.1 |  |
| MT344948.1 | KF514407.1 |  |
| MT345875.1 | FJ882954.1 |  |
| MT325638.1 | FJ882929.1 |  |
| MT325637.1 | JX162087.1 |  |
| MT325633.1 | AY463059.1 |  |
| MT325632.1 | AY351680.1 |  |
| MT325631.1 | AY463060.1 |  |
| MT325630.1 | AY559097.1 |  |
| MT325627.1 | AY545915.1 |  |
| MT325626.1 |  |  |
| MT325616.1 |  |  |
| MT325611.1 |  |  |
| MT325605.1 |  |  |
| MT325603.1 |  |  |
| MT325601.1 |  |  |
| MT325600.1 |  |  |
| MT325599.1 |  |  |
| MT325598.1 |  |  |
| MT325594.1 |  |  |
| MT325590.1 |  |  |
| MT325583.1 |  |  |
| MT325582.1 |  |  |
| MT325580.1 |  |  |
| MT325578.1 |  |  |
| MT325565.1 |  |  |
| MT325561.1 |  |  |
| MT322422.1 |  |  |
| MT304488.1 |  |  |
| MT304486.1 |  |  |
| MT293219.1 |  |  |
| MT276326.1 |  |  |
| MT262915.1 |  |  |
| MT262899.1 |  |  |
| MT262896.1 |  |  |
| MT258379.1 |  |  |
| MT126808.1 |  |  |
| MT066176.1 |  |  |
| MT066175.1 |  |  |
| MT252794.1 |  |  |
| MT358695.1 |  |  |
| MT358649.1 |  |  |
| MT344962.1 |  |  |
| MT345834.1 |  |  |
| MT326092.1 |  |  |
| MT322402.1 |  |  |
| MT293204.1 |  |  |
| MT251976.1 |  |  |
| LC528232.1 |  |  |
| MT374103.1 |  |  |
| MT358401.1 |  |  |
| MT358744.1 |  |  |
| MT358742.1 |  |  |
| MT344959.1 |  |  |
| MT344949.1 |  |  |
| MT344947.1 |  |  |
| MT345840.1 |  |  |
| MT325602.1 |  |  |
| MT325585.1 |  |  |
| MT325584.1 |  |  |
| MT324684.1 |  |  |
| MT246459.1 |  |  |
| MT192759.1 |  |  |
| LC528233.1 |  |  |
| MT380732.1 |  |  |
| MT380728.1 |  |  |
| MT350275.1 |  |  |
| MT350268.1 |  |  |
| MT344960.1 |  |  |
| MT334546.1 |  |  |
| MT325639.1 |  |  |
| MT325636.1 |  |  |
| MT325635.1 |  |  |
| MT325629.1 |  |  |
| MT325628.1 |  |  |
| MT325587.1 |  |  |
| MT325586.1 |  |  |
| MT325577.1 |  |  |
| MT325574.1 |  |  |
| MT325568.1 |  |  |
| MT326052.1 |  |  |
| MT304490.1 |  |  |
| MT276323.1 |  |  |
| MT263468.1 |  |  |
| MT385443.1 |  |  |
| MT252784.1 |  |  |
| MT252677.1 |  |  |
| MT350251.1 |  |  |
| MT325612.1 |  |  |
| MT295465.1 |  |  |
| MT258383.1 |  |  |
| MT259230.1 |  |  |
| MT152824.1 |  |  |
| MT385458.1 |  |  |
| MT252793.1 |  |  |
| MT358743.1 |  |  |
| MT350240.1 |  |  |
| MT345869.1 |  |  |
| MT325564.1 |  |  |
| MT304478.1 |  |  |
| MT259231.1 |  |  |
| MT123292.2 |  |  |
| MT066156.1 |  |  |
| MT385442.1 |  |  |
| MT385437.1 |  |  |
| MT114416.1 |  |  |
| MT350253.1 |  |  |
| MT350271.1 |  |  |
| MT350269.1 |  |  |
| MT344954.1 |  |  |
| MT325622.1 |  |  |
| MT325595.1 |  |  |
| MT039874.1 |  |  |
| MT304484.1 |  |  |
| MT304479.1 |  |  |
| MT291827.1 |  |  |
| MT276329.1 |  |  |
| MT276327.1 |  |  |
| MT259229.1 |  |  |
| MT252763.1 |  |  |
| MT375432.1 |  |  |
| MT114419.1 |  |  |
| MT345882.1 |  |  |
| MT328034.1 |  |  |
| MT325610.1 |  |  |
| MT325589.1 |  |  |
| MT291831.1 |  |  |
| MT093631.2 |  |  |
| MT344944.1 |  |  |
| MT325597.1 |  |  |
| MT325569.1 |  |  |
| MT263459.1 |  |  |
| MT123293.2 |  |  |
| MT385460.1 |  |  |
| MT385449.1 |  |  |
| MT380733.1 |  |  |
| MT344945.1 |  |  |
| MT345806.1 |  |  |
| MT325624.1 |  |  |
| MT325623.1 |  |  |
| MT325606.1 |  |  |
| MT325570.1 |  |  |
| MT326167.1 |  |  |
| MT326100.1 |  |  |
| MT304480.1 |  |  |
| MT291828.1 |  |  |
| MT385440.1 |  |  |
| MT252707.1 |  |  |
| MT358716.1 |  |  |
| MT344953.1 |  |  |
| MT345868.1 |  |  |
| MT345841.1 |  |  |
| MT325604.1 |  |  |
| MT325596.1 |  |  |
| MT304491.1 |  |  |
| MT304477.1 |  |  |
| MT293159.1 |  |  |
| MT385445.1 |  |  |
| MT380729.1 |  |  |
| MT375430.1 |  |  |
| MT358718.1 |  |  |
| MT350252.1 |  |  |
| MT334535.1 |  |  |
| MT304489.1 |  |  |
| MT259269.1 |  |  |
| MT246474.1 |  |  |
| MT385444.1 |  |  |
| MT380734.1 |  |  |
| MT380730.1 |  |  |
| MT252769.1 |  |  |
| MT375463.1 |  |  |
| MT345888.1 |  |  |
| MT325625.1 |  |  |
| MT325607.1 |  |  |
| MT325588.1 |  |  |
| MT325581.1 |  |  |
| MT325575.1 |  |  |
| MT325572.1 |  |  |
| MT325571.1 |  |  |
| MT322423.1 |  |  |
| MT252764.1 |  |  |
| MT375431.1 |  |  |
| MT372481.1 |  |  |
| MT358722.1 |  |  |
| MT358706.1 |  |  |
| MT345873.1 |  |  |
| MT328033.1 |  |  |
| MT263438.1 |  |  |
| MT263404.1 |  |  |
| MT246450.1 |  |  |
| MT385448.1 |  |  |
| MT385446.1 |  |  |
| MT344963.1 |  |  |
| MT325567.1 |  |  |
| MT291834.1 |  |  |
| MT293210.1 |  |  |
| MT263437.1 |  |  |
| MT263421.1 |  |  |
| MT259226.1 |  |  |
| MT385452.1 |  |  |
| MT252716.1 |  |  |
| MT375482.1 |  |  |
| MT375440.1 |  |  |
| MT350254.1 |  |  |
| MT350245.1 |  |  |
| MT350244.1 |  |  |
| MT322395.1 |  |  |
| MT259273.1 |  |  |
| MT259236.1 |  |  |
| MT252806.1 |  |  |
| MT252774.1 |  |  |
| MT358748.1 |  |  |
| MT358696.1 |  |  |
| MT345803.1 |  |  |
| MT325608.1 |  |  |
| MT325573.1 |  |  |
| MT326153.1 |  |  |
| MT322408.1 |  |  |
| MT293202.1 |  |  |
| MT293181.1 |  |  |
| MT263399.1 |  |  |
| MT246481.1 |  |  |
| MT246477.1 |  |  |
| MT385441.1 |  |  |
| MT252719.1 |  |  |
| MT375483.1 |  |  |
| MT326132.1 |  |  |
| MT322404.1 |  |  |
| MT293195.1 |  |  |
| MT293192.1 |  |  |
| MT252688.1 |  |  |
| MT358741.1 |  |  |
| MT345871.1 |  |  |
| MT326065.1 |  |  |
| MT293208.1 |  |  |
| MT263458.1 |  |  |
| MT263446.1 |  |  |
| MT263435.1 |  |  |
| MT263406.1 |  |  |
| MT259251.1 |  |  |
| MT259228.1 |  |  |
| MT385455.1 |  |  |
| MT252807.1 |  |  |
| MT252804.1 |  |  |
| MT375478.1 |  |  |
| MT358719.1 |  |  |
| MT326095.1 |  |  |
| MT326088.1 |  |  |
| MT293176.1 |  |  |
| MT276597.1 |  |  |
| MT263403.1 |  |  |
| MT263391.1 |  |  |
| MT259263.1 |  |  |
| MT246461.1 |  |  |
| MT385457.1 |  |  |
| MT385456.1 |  |  |
| MT385451.1 |  |  |
| MT350250.1 |  |  |
| MT350247.1 |  |  |
| MT326150.1 |  |  |
| MT326129.1 |  |  |
| MT326118.1 |  |  |
| MT326116.1 |  |  |
| MT326048.1 |  |  |
| MT322394.1 |  |  |
| MT293218.1 |  |  |
| MT259260.1 |  |  |
| MT252782.1 |  |  |
| MT252742.1 |  |  |
| MT358723.1 |  |  |
| MT358684.1 |  |  |
| MT344958.1 |  |  |
| MT344956.1 |  |  |
| MT344955.1 |  |  |
| MT326067.1 |  |  |
| MT326066.1 |  |  |
| MT322406.1 |  |  |
| MT385450.1 |  |  |
| MT252746.1 |  |  |
| MT375470.1 |  |  |
| MT375455.1 |  |  |
| MT358667.1 |  |  |
| MT345809.1 |  |  |
| MT345805.1 |  |  |
| MT326069.1 |  |  |
| MT326063.1 |  |  |
| MT293187.1 |  |  |
| MT293177.1 |  |  |
| MT276598.1 |  |  |
| MT263418.1 |  |  |
| MT246471.1 |  |  |
| MT375445.1 |  |  |
| MT326164.1 |  |  |
| MT326103.1 |  |  |
| MT322415.1 |  |  |
| MT322412.1 |  |  |
| MT293212.1 |  |  |
| MT293196.1 |  |  |
| MT263447.1 |  |  |
| MT375458.1 |  |  |
| MT375438.1 |  |  |
| MT358647.1 |  |  |
| MT350249.1 |  |  |
| MT344957.1 |  |  |
| MT345887.1 |  |  |
| MT345816.1 |  |  |
| MT293172.1 |  |  |
| MT246475.1 |  |  |
| MT385447.1 |  |  |
| MT252805.1 |  |  |
| MT252767.1 |  |  |
| MT375452.1 |  |  |
| MT345858.1 |  |  |
| MT345835.1 |  |  |
| MT326106.1 |  |  |
| MT322411.1 |  |  |
| MT291836.1 |  |  |
| MT293179.1 |  |  |
| MT246476.1 |  |  |
| MT385439.1 |  |  |
| MT358747.1 |  |  |
| MT345833.1 |  |  |
| MT293213.1 |  |  |
| MT263463.1 |  |  |
| MT259244.1 |  |  |
| MT246469.1 |  |  |
| MT385463.1 |  |  |
| MT385459.1 |  |  |
| MT345867.1 |  |  |
| MT345861.1 |  |  |
| MT293161.1 |  |  |
| MT251975.1 |  |  |
| MT375441.1 |  |  |
| MT358735.1 |  |  |
| MT358698.1 |  |  |
| MT358661.1 |  |  |
| MT350241.1 |  |  |
| MT350237.1 |  |  |
| MT345865.1 |  |  |
| MT293225.1 |  |  |
| MT293220.1 |  |  |
| MT293198.1 |  |  |
| MT293189.1 |  |  |
| MT293166.1 |  |  |
| MT259227.1 |  |  |
| MT252800.1 |  |  |
| MT252745.1 |  |  |
| MT358646.1 |  |  |
| MT350236.1 |  |  |
| MT326140.1 |  |  |
| MT322420.1 |  |  |
| MT293183.1 |  |  |
| MT263444.1 |  |  |
| MT252810.1 |  |  |
| MT252747.1 |  |  |
| MT358657.1 |  |  |
| MT345815.1 |  |  |
| MT326127.1 |  |  |
| MT326099.1 |  |  |
| MT326068.1 |  |  |
| MT263440.1 |  |  |
| MT263432.1 |  |  |
| MT259271.1 |  |  |
| MT252710.1 |  |  |
| MT358687.1 |  |  |
| MT345878.1 |  |  |
| MT345872.1 |  |  |
| MT326154.1 |  |  |
| MT326055.1 |  |  |
| MT322407.1 |  |  |
| MT293216.1 |  |  |
| MT293207.1 |  |  |
| MT263419.1 |  |  |
| MT263417.1 |  |  |
| MT259286.1 |  |  |
| MT050493.1 |  |  |
| MT358734.1 |  |  |
| MT358662.1 |  |  |
| MT350243.1 |  |  |
| MT345853.1 |  |  |
| MT326137.1 |  |  |
| MT326030.1 |  |  |
| MT293178.1 |  |  |
| MT252792.1 |  |  |
| MT252713.1 |  |  |
| MT358703.1 |  |  |
| MT326096.1 |  |  |
| MT326029.1 |  |  |
| MT246455.1 |  |  |
| MT012098.1 |  |  |
| MT385464.1 |  |  |
| MT252795.1 |  |  |
| MT372480.1 |  |  |
| MT358721.1 |  |  |
| MT345827.1 |  |  |
| MT345825.1 |  |  |
| MT326119.1 |  |  |
| MT263445.1 |  |  |
| MT252798.1 |  |  |
| MT375437.1 |  |  |
| MT345836.1 |  |  |
| MT345828.1 |  |  |
| MT345811.1 |  |  |
| MT263074.1 |  |  |
| MT259277.1 |  |  |
| MT259257.1 |  |  |
| MT246470.1 |  |  |
| MT385465.1 |  |  |
| MT385462.1 |  |  |
| MT385453.1 |  |  |
| MT291835.2 |  |  |
| MT358671.1 |  |  |
| MT350257.1 |  |  |
| MT350255.1 |  |  |
| MT345886.1 |  |  |
| MT326027.1 |  |  |
| MT322421.1 |  |  |
| MT322416.1 |  |  |
| MT259237.1 |  |  |
| MT385461.1 |  |  |
| MT358710.1 |  |  |
| MT358700.1 |  |  |
| MT358688.1 |  |  |
| MT350246.1 |  |  |
| MT345885.1 |  |  |
| MT322396.1 |  |  |
| MT293182.1 |  |  |
| MT293171.1 |  |  |
| MT263439.1 |  |  |
| MT246489.1 |  |  |
| MT246486.1 |  |  |
| MT246484.1 |  |  |
| MT252741.1 |  |  |
| MT358730.1 |  |  |
| MT358658.1 |  |  |
| MT345798.1 |  |  |
| MT251974.1 |  |  |
| MT251973.1 |  |  |
| MT246490.1 |  |  |
| MT252770.1 |  |  |
| MT252728.1 |  |  |
| MT358693.1 |  |  |
| MT326042.1 |  |  |
| MT263465.1 |  |  |
| MT263413.1 |  |  |
| MT263411.1 |  |  |
| MT263410.1 |  |  |
| MT252785.1 |  |  |
| MT252739.1 |  |  |
| MT375460.1 |  |  |
| MT350242.1 |  |  |
| MT345854.1 |  |  |
| MT345830.1 |  |  |
| MT326162.1 |  |  |
| MT326097.1 |  |  |
| MT326061.1 |  |  |
| MT293186.1 |  |  |
| MT263443.1 |  |  |
| MT263431.1 |  |  |
| MT259248.1 |  |  |
| MT385468.1 |  |  |
| MT385454.1 |  |  |
| MT252717.1 |  |  |
| MT252697.1 |  |  |
| MT263467.1 |  |  |
| MT385469.1 |  |  |
| MT375466.1 |  |  |
| MT345802.1 |  |  |
| MT326089.1 |  |  |
| MT326086.1 |  |  |
| MT326056.1 |  |  |
| MT326023.1 |  |  |
| MT263433.1 |  |  |
| MT246487.1 |  |  |
| MT385474.1 |  |  |
| MT385473.1 |  |  |
| MT385470.1 |  |  |
| MT252698.1 |  |  |
| MT358739.1 |  |  |
| MT358733.1 |  |  |
| MT358670.1 |  |  |
| MT345849.1 |  |  |
| MT293160.1 |  |  |
| MT345879.1 |  |  |
| MT345846.1 |  |  |
| MT326087.1 |  |  |
| MT293201.1 |  |  |
| MT259275.1 |  |  |
| MT252682.1 |  |  |
| MT358655.1 |  |  |
| MT350248.1 |  |  |
| MT293222.1 |  |  |
| MT358659.1 |  |  |
| MT259246.1 |  |  |
| MT240479.1 |  |  |
| MT385466.1 |  |  |
| MT252735.1 |  |  |
| MT358656.1 |  |  |
| MT263425.1 |  |  |
| MT263402.1 |  |  |
| MT308702.1 |  |  |
| MT291832.1 |  |  |
| MT293215.1 |  |  |
| MT263400.1 |  |  |
| MT252799.1 |  |  |
| MT358738.1 |  |  |
| MT358717.1 |  |  |
| MT358653.1 |  |  |
| MT293162.1 |  |  |
| MT263430.1 |  |  |
| MT246451.1 |  |  |
| MT226610.1 |  |  |
| MT252781.1 |  |  |
| MT358711.1 |  |  |
| MT358701.1 |  |  |
| MT358678.1 |  |  |
| MT327745.1 |  |  |
| MT293211.1 |  |  |
| MT259281.1 |  |  |
| MT246457.1 |  |  |
| MT192765.1 |  |  |
| MT358732.1 |  |  |
| MT358709.1 |  |  |
| MT358654.1 |  |  |
| MT326113.1 |  |  |
| MT326081.1 |  |  |
| MT322403.1 |  |  |
| MT300186.2 |  |  |
| MT375451.1 |  |  |
| MT358713.1 |  |  |
| MT345883.1 |  |  |
| MT345826.1 |  |  |
| MT326051.1 |  |  |
| MT293209.1 |  |  |
| MT293165.1 |  |  |
| MT263469.1 |  |  |
| MT259264.1 |  |  |
| MT375461.1 |  |  |
| MT358737.1 |  |  |
| MT358691.1 |  |  |
| MT358690.1 |  |  |
| MT358648.1 |  |  |
| MT326120.1 |  |  |
| MT263450.1 |  |  |
| MT246488.1 |  |  |
| MT252691.1 |  |  |
| MT358668.1 |  |  |
| MT345832.1 |  |  |
| MT326070.1 |  |  |
| MT322398.1 |  |  |
| MT252738.1 |  |  |
| MT252729.1 |  |  |
| MT358712.1 |  |  |
| MT358682.1 |  |  |
| MT358644.1 |  |  |
| MT326169.1 |  |  |
| MT326128.1 |  |  |
| MT326074.1 |  |  |
| MT291833.1 |  |  |
| MT263464.1 |  |  |
| MT263414.1 |  |  |
| MT251972.1 |  |  |
| MT252733.1 |  |  |
| MT358728.1 |  |  |
| MT358665.1 |  |  |
| MT358651.1 |  |  |
| MT326078.1 |  |  |
| MT263423.1 |  |  |
| MT259261.1 |  |  |
| MT072688.1 |  |  |
| MT385471.1 |  |  |
| MT252757.1 |  |  |
| MT358660.1 |  |  |
| MT345881.1 |  |  |
| MT345870.1 |  |  |
| MT320891.2 |  |  |
| MT291830.1 |  |  |
| MT263452.1 |  |  |
| MT263436.1 |  |  |
| MT259267.1 |  |  |
| MT358689.1 |  |  |
| MT326082.1 |  |  |
| MT326076.1 |  |  |
| MT326075.1 |  |  |
| MT252788.1 |  |  |
| MT252778.1 |  |  |
| MT358729.1 |  |  |
| MT358669.1 |  |  |
| MT350256.1 |  |  |
| MT345829.1 |  |  |
| MT326040.1 |  |  |
| MT293190.1 |  |  |
| MT263420.1 |  |  |
| MT252772.1 |  |  |
| MT358746.1 |  |  |
| MT358724.1 |  |  |
| MT358699.1 |  |  |
| MT358666.1 |  |  |
| MT358652.1 |  |  |
| MT326134.1 |  |  |
| MT293156.1 |  |  |
| MT263392.1 |  |  |
| MT385472.1 |  |  |
| MT252679.1 |  |  |
| MT358664.1 |  |  |
| MT326159.1 |  |  |
| MT326133.1 |  |  |
| MT259256.1 |  |  |
| MT358683.1 |  |  |
| MT358645.1 |  |  |
| MT345817.1 |  |  |
| MT326105.1 |  |  |
| MT326028.1 |  |  |
| MT252736.1 |  |  |
| MT358681.1 |  |  |
| MT345874.1 |  |  |
| MT345856.1 |  |  |
| MT293205.1 |  |  |
| MT293200.1 |  |  |
| MT252775.1 |  |  |
| MT350238.1 |  |  |
| MT326058.1 |  |  |
| MT258382.1 |  |  |
| MT358677.1 |  |  |
| MT358672.1 |  |  |
| MT326148.1 |  |  |
| MT263449.1 |  |  |
| MT252686.1 |  |  |
| MT326180.1 |  |  |
| MT326093.1 |  |  |
| MT308703.1 |  |  |
| MT291826.1 |  |  |
| MT263405.1 |  |  |
| MT252694.1 |  |  |
| MT252685.1 |  |  |
| MT252678.1 |  |  |
| MT293224.1 |  |  |
| MT263415.1 |  |  |
| MT259282.1 |  |  |
| MT246482.1 |  |  |
| MT246479.1 |  |  |
| MT246464.1 |  |  |
| MT252723.1 |  |  |
| MT263422.1 |  |  |
| MT251977.1 |  |  |
| MT385467.1 |  |  |
| MT326084.1 |  |  |
| MT263416.1 |  |  |
| MT259249.1 |  |  |
| MT345859.1 |  |  |
| MT293173.1 |  |  |
| MT263454.1 |  |  |
| MT259245.1 |  |  |
| MT246468.1 |  |  |
| MT252690.1 |  |  |
| MT263412.1 |  |  |
| MT251980.1 |  |  |
| MT252749.1 |  |  |
| MT375465.1 |  |  |
| MT375472.1 |  |  |
| MT326104.1 |  |  |
| MT252680.1 |  |  |
| MT326071.1 |  |  |
| MT263424.1 |  |  |
| MT259253.1 |  |  |
| MT246453.1 |  |  |
| MT263442.1 |  |  |
| MT252696.1 |  |  |
| MT344951.1 |  |  |
| MT344950.1 |  |  |
| MT358680.1 |  |  |
| MT345866.1 |  |  |
| MT263408.1 |  |  |
| MT252761.1 |  |  |
| MT345880.1 |  |  |
| MT326191.1 |  |  |
| MT246449.1 |  |  |
| MT252684.1 |  |  |
| MT358714.1 |  |  |
| MT344952.1 |  |  |
| MT326125.1 |  |  |
| MT251979.1 |  |  |
| MT252689.1 |  |  |
| MT358697.1 |  |  |
| MT263448.1 |  |  |
| MT326112.1 |  |  |
| MT326049.1 |  |  |
| MT253696.1 |  |  |
| MT358731.1 |  |  |
| MT308704.1 |  |  |
| MT345876.1 |  |  |
| MT077125.1 |  |  |
| MT259285.1 |  |  |
| MT253706.1 |  |  |
| MT358663.1 |  |  |
| MT358650.1 |  |  |
| MT263457.1 |  |  |
| MT252740.1 |  |  |
| MT252699.1 |  |  |
| MT252687.1 |  |  |
| MT370891.1 |  |  |
| MT252758.1 |  |  |
| MT291829.1 |  |  |
| MT252700.1 |  |  |
| MT326185.1 |  |  |
| MT292572.1 |  |  |
| MT252756.1 |  |  |
| MT252703.1 |  |  |
| MT263434.1 |  |  |
| MT326039.1 |  |  |
| MT252726.1 |  |  |
| MT375433.1 |  |  |
| MT345801.1 |  |  |
| MT326090.1 |  |  |
| MT252705.1 |  |  |
| MT252704.1 |  |  |
| MT326135.1 |  |  |
| MT326130.1 |  |  |
| MT292569.1 |  |  |
| MT246472.1 |  |  |
| MT252695.1 |  |  |
| MT358673.1 |  |  |
| MT246473.1 |  |  |
| MT252768.1 |  |  |
| MT326085.1 |  |  |
| MT256924.2 |  |  |
| MT292575.1 |  |  |
| MT259266.1 |  |  |
| MT233523.1 |  |  |
| MT292570.1 |  |  |
| MT198652.2 |  |  |
| MT292573.1 |  |  |
| MT358679.1 |  |  |
| MT345855.1 |  |  |
| MT326111.1 |  |  |
| MT293188.1 |  |  |
| MT233519.1 |  |  |
| MT252712.1 |  |  |
| MT292574.1 |  |  |
| MT252708.1 |  |  |
| MT252706.1 |  |  |
| MT370861.1 |  |  |
| MT370866.1 |  |  |
| MT259252.1 |  |  |
| MT358692.1 |  |  |
| MT263456.1 |  |  |
| MT375480.1 |  |  |
| MT375443.1 |  |  |
| MT370906.1 |  |  |
| MT370996.1 |  |  |
| MT345824.1 |  |  |
| MT293175.1 |  |  |
| MT370919.1 |  |  |
| MT375457.1 |  |  |
| MT370965.1 |  |  |
| MT263462.1 |  |  |
| MT263428.1 |  |  |
| MT345844.1 |  |  |
| MT252725.1 |  |  |
| MT375476.1 |  |  |
| MT326041.1 |  |  |
| MT370903.1 |  |  |
| MT252681.1 |  |  |
| MT252709.1 |  |  |
| MT263383.1 |  |  |
| MT259268.1 |  |  |
| MT252823.1 |  |  |
| MT371574.1 |  |  |
| MT371573.1 |  |  |
| MT371571.1 |  |  |
| MT326160.1 |  |  |
| MT375434.1 |  |  |
| MT326035.1 |  |  |
| MT259243.1 |  |  |
| MT371570.1 |  |  |
| MT326046.1 |  |  |
| MT252803.1 |  |  |
| MT259274.1 |  |  |
| MT370874.1 |  |  |
| MT371572.1 |  |  |
| MT326036.1 |  |  |
| MT375468.1 |  |  |
| MT370895.1 |  |  |
| MT345812.1 |  |  |
| MT259235.1 |  |  |
| MT293164.1 |  |  |
| MT375442.1 |  |  |
| MT326053.1 |  |  |
| MT263390.1 |  |  |
| MT370944.1 |  |  |
| MT252721.1 |  |  |
| MT293169.1 |  |  |
| MT252822.1 |  |  |
| MT370937.1 |  |  |
| MT370925.1 |  |  |
| MT370909.1 |  |  |
| MT252715.1 |  |  |
| MT252683.1 |  |  |
| MT370888.1 |  |  |
| MT326166.1 |  |  |
| MT370966.1 |  |  |
| MT370950.1 |  |  |
| MT326152.1 |  |  |
| MT370872.1 |  |  |
| MT370835.1 |  |  |
| MT370911.1 |  |  |
| MT370905.1 |  |  |
| MT370853.1 |  |  |
| MT326138.1 |  |  |
| MT370851.1 |  |  |
| MT293170.1 |  |  |
| MT370948.1 |  |  |
| MT345814.1 |  |  |
| MT293158.1 |  |  |
| MT263441.1 |  |  |
| MT375467.1 |  |  |
| MT370985.1 |  |  |
| MT370964.1 |  |  |
| MT370934.1 |  |  |
| MT370899.1 |  |  |
| MT370849.1 |  |  |
| MT370843.1 |  |  |
| MT326158.1 |  |  |
| MT326124.1 |  |  |
| MT370954.1 |  |  |
| MT326149.1 |  |  |
| MT371028.1 |  |  |
| MT371013.1 |  |  |
| MT371009.1 |  |  |
| MT371008.1 |  |  |
| MT371001.1 |  |  |
| MT370980.1 |  |  |
| MT370920.1 |  |  |
| MT370912.1 |  |  |
| MT370901.1 |  |  |
| MT370897.1 |  |  |
| MT370886.1 |  |  |
| MT370877.1 |  |  |
| MT370865.1 |  |  |
| MT370846.1 |  |  |
| MT370845.1 |  |  |
| MT326184.1 |  |  |
| MT371034.1 |  |  |
| MT371033.1 |  |  |
| MT371026.1 |  |  |
| MT371006.1 |  |  |
| MT370975.1 |  |  |
| MT370889.1 |  |  |
| MT370881.1 |  |  |
| MT370869.1 |  |  |
| MT370857.1 |  |  |
| MT370834.1 |  |  |
| MT345822.1 |  |  |
| MT370971.1 |  |  |
| MT371036.1 |  |  |
| MT371018.1 |  |  |
| MT370994.1 |  |  |
| MT370915.1 |  |  |
| MT370902.1 |  |  |
| MT370890.1 |  |  |
| MT370876.1 |  |  |
| MT370856.1 |  |  |
| MT370838.1 |  |  |
| MT370836.1 |  |  |
| MT371015.1 |  |  |
| MT371012.1 |  |  |
| MT370957.1 |  |  |
| MT370953.1 |  |  |
| MT370907.1 |  |  |
| MT370892.1 |  |  |
| MT370885.1 |  |  |
| MT370962.1 |  |  |
| MT326123.1 |  |  |
| MT252783.1 |  |  |
| MT371027.1 |  |  |
| MT371016.1 |  |  |
| MT370932.1 |  |  |
| MT370914.1 |  |  |
| MT370894.1 |  |  |
| MT370839.1 |  |  |
| MT375456.1 |  |  |
| MT371019.1 |  |  |
| MT370958.1 |  |  |
| MT370913.1 |  |  |
| MT370880.1 |  |  |
| MT370870.1 |  |  |
| MT370854.1 |  |  |
| MT370844.1 |  |  |
| MT326151.1 |  |  |
| MT371007.1 |  |  |
| MT370908.1 |  |  |
| MT370896.1 |  |  |
| MT370883.1 |  |  |
| MT370859.1 |  |  |
| MT371023.1 |  |  |
| MT371000.1 |  |  |
| MT370998.1 |  |  |
| MT370984.1 |  |  |
| MT370939.1 |  |  |
| MT370933.1 |  |  |
| MT370875.1 |  |  |
| MT370864.1 |  |  |
| MT370850.1 |  |  |
| MT370837.1 |  |  |
| MT345860.1 |  |  |
| MT370981.1 |  |  |
| MT370967.1 |  |  |
| MT370930.1 |  |  |
| MT370916.1 |  |  |
| MT370882.1 |  |  |
| MT370858.1 |  |  |
| MT371017.1 |  |  |
| MT370970.1 |  |  |
| MT370947.1 |  |  |
| MT370842.1 |  |  |
| MT371035.1 |  |  |
| MT370940.1 |  |  |
| MT370935.1 |  |  |
| MT370988.1 |  |  |
| MT370987.1 |  |  |
| MT370862.1 |  |  |
| MT326094.1 |  |  |
| MT371029.1 |  |  |
| MT371002.1 |  |  |
| MT370904.1 |  |  |
| MT370898.1 |  |  |
| MT375454.1 |  |  |
| MT375450.1 |  |  |
| MT370993.1 |  |  |
| MT370979.1 |  |  |
| MT370868.1 |  |  |
| MT370860.1 |  |  |
| MT370833.1 |  |  |
| MT370863.1 |  |  |
| MT293191.1 |  |  |
| MT370986.1 |  |  |
| MT370931.1 |  |  |
| MT370871.1 |  |  |
| MT370841.1 |  |  |
| MT358708.1 |  |  |
| MT371032.1 |  |  |
| MT371022.1 |  |  |
| MT370960.1 |  |  |
| MT370949.1 |  |  |
| MT371038.1 |  |  |
| MT370946.1 |  |  |
| MT263382.1 |  |  |
| MT252787.1 |  |  |
| MT370961.1 |  |  |
| MT370927.1 |  |  |
| MT326054.1 |  |  |
| MT370917.1 |  |  |
| MT246456.1 |  |  |
| MT252693.1 |  |  |
| MT370977.1 |  |  |
| MT370831.1 |  |  |
| MT263388.1 |  |  |
| MT370956.1 |  |  |
| MT370942.1 |  |  |
| MT358720.1 |  |  |
| MT370991.1 |  |  |
| MT326091.1 |  |  |
| MT263394.1 |  |  |
| MT252737.1 |  |  |
| MT246485.1 |  |  |
| MT326136.1 |  |  |
| MT259241.1 |  |  |
| MT345842.1 |  |  |
| MT345848.1 |  |  |
| MT370972.1 |  |  |
| MT345808.1 |  |  |
| MT326083.1 |  |  |
| MT252773.1 |  |  |
| MT326024.1 |  |  |
| MT293199.1 |  |  |
| MT293184.1 |  |  |
| MT259287.1 |  |  |
| MT370848.1 |  |  |
| MT293168.1 |  |  |
| MT345862.1 |  |  |
| MT375477.1 |  |  |
| MT263455.1 |  |  |
| MT293174.1 |  |  |
| MT252801.1 |  |  |
| MT375459.1 |  |  |
| MT263451.1 |  |  |
| MT263386.1 |  |  |
| MT293167.1 |  |  |
| MT375471.1 |  |  |
| MT293185.1 |  |  |
| MT326181.1 |  |  |
| MT259258.1 |  |  |
| MT259250.1 |  |  |
| MT259247.1 |  |  |
| MT293194.1 |  |  |
| MT252811.1 |  |  |
| MT345847.1 |  |  |
| MT326121.1 |  |  |
| MT263398.1 |  |  |
| MT259280.1 |  |  |
| MT252809.1 |  |  |
| MT293197.1 |  |  |
| MT345821.1 |  |  |
| MT293206.1 |  |  |
| MT345851.1 |  |  |
| MT345799.1 |  |  |
| MT252802.1 |  |  |
| MT233522.1 |  |  |
| MT345823.1 |  |  |
| MT326161.1 |  |  |
| MT263384.1 |  |  |
| MT293163.1 |  |  |
| MT259239.1 |  |  |
| MT326173.1 |  |  |
| MT345838.1 |  |  |
| MT263426.1 |  |  |
| MT263387.1 |  |  |
| MT345857.1 |  |  |
| MT374101.1 |  |  |
| MT385486.1 |  |  |
| MT339043.1 |  |  |
| MT370976.1 |  |  |
| MT322414.1 |  |  |
| MT371568.1 |  |  |
| MT370878.1 |  |  |
| MT375444.1 |  |  |
| MT246458.1 |  |  |
| MT358726.1 |  |  |
| MT375435.1 |  |  |
| MT252701.1 |  |  |
| MT263453.1 |  |  |
| MT293180.1 |  |  |
| MT358707.1 |  |  |
| MT371569.1 |  |  |
| MT370952.1 |  |  |
| MT385478.1 |  |  |
| MT370852.1 |  |  |
| MT370997.1 |  |  |
| MT358674.1 |  |  |
| MT252730.1 |  |  |
| MT334557.1 |  |  |
| MT375462.1 |  |  |
| MT334539.1 |  |  |
| MT334536.1 |  |  |
| MT334523.1 |  |  |
| MT334531.1 |  |  |
| MT334537.1 |  |  |
| MT334561.1 |  |  |
| MT334526.1 |  |  |
| MT322400.1 |  |  |
| MT375449.1 |  |  |
| MT385476.1 |  |  |
| MT385475.1 |  |  |
| MT385487.1 |  |  |
| MT385480.1 |  |  |
| MT259284.1 |  |  |
| MT358685.1 |  |  |
| MT358725.1 |  |  |
| MT345843.1 |  |  |
| MT345852.1 |  |  |
| MT345884.1 |  |  |
| MT385477.1 |  |  |
| MT385482.1 |  |  |
| MT385481.1 |  |  |
| MT385479.1 |  |  |
| MT345850.1 |  |  |
| MT372482.1 |  |  |
| MT339040.1 |  |  |
| MT358745.1 |  |  |
| MT385491.1 |  |  |
| MT375436.1 |  |  |
| MT385483.1 |  |  |
| MT375429.1 |  |  |
| MT385488.1 |  |  |
| MT385489.1 |  |  |
| MT385484.1 |  |  |
| MT375469.1 |  |  |
| MT385490.1 |  |  |
| MT385492.1 |  |  |
| MT375448.1 |  |  |
| MT385485.1 |  |  |
| MT375428.1 |  |  |
| MT385495.1 |  |  |
| MT385493.1 |  |  |
| MT385496.1 |  |  |
| MT385497.1 |  |  |
| MT385494.1 |  |  |

Figure S1. Simple Neighbor-joining tree for nine different complete coronavirus genomes. Note that this tree is only for illustrative purposes.


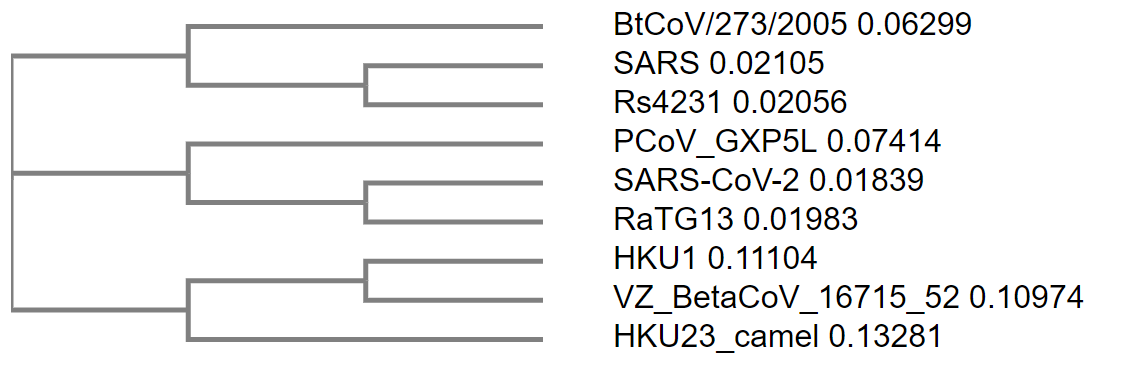


Table S2. Coordinates of SARS-CoV-2 G to U transversions. The coordinates and gene annotations are based on SARS-CoV-2 Reference Sequences: NC_045512.2. First nucleotide has coordinate 1.

| Coodinate | Type | Nucleotide/Codon change |
| --- | --- | --- |
| 53 | Nongene | G to U |
| 88 | Nongene | G to U |
| 90 | Nongene | G to U |
| 101 | Nongene | G to U |
| 198 | Nongene | G to U |
| 199 | Nongene | G to U |
| 208 | Nongene | G to U |
| 219 | Nongene | G to U |
| 261 | Nongene | G to U |
| 332 | Nonsynonymous | GUU to UUU |
| 598 | Synonymous | GUG to GUU |
| 625 | Nonsynonymous | AAG to AAU |
| 1148 | Nonsynonymous | GGC to UGC |
| 1580 | Nonsynonymous | GUU to UUU |
| 1895 | Nonsynonymous | GUA to UUA |
| 2632 | Nonsynonymous | AUG to AUU |
| 2764 | Synonymous | GUG to GUU |
| 2782 | Synonymous | GUG to GUU |
| 2951 | Nonsynonymous | GAU to UAU |
| 2957 | Nonsynonymous | GAU to UAU |
| 3231 | Nonsynonymous | GGU to GUU |
| 3251 | Nonsynonymous | GAC to UAC |
| 3259 | Nonsynonymous | CAG to CAU |
| 3286 | Nonsynonymous | GAG to GAU |
| 3871 | Nonsynonymous | AAG to AAU |
| 3947 | Nonsynonymous | GAU to UAU |
| 3955 | Nonsynonymous | AAG to AAU |
| 4148 | Nonsynonymous | GUU to UUU |
| 4201 | Nonsynonymous | AUG to AUU |
| 4255 | Synonymous | CCG to CCU |
| 4288 | Nonsynonymous | GAG to GAU |
| 4975 | Synonymous | GUG to GUU |
| 5062 | Nonsynonymous | UUG to UUU |
| 5572 | Nonsynonymous | AUG to AUU |
| 5716 | Nonsynonymous | AAG to AAU |
| 6819 | Nonsynonymous | AGU to AUU |
| 7675 | Synonymous | GCG to GCU |
| 7936 | Synonymous | GCG to GCU |
| 8102 | Nonsynonymous | GUU to UUU |
| 9049 | Nonsynonymous | AAG to AAU |
| 9479 | Nonsynonymous | GGU to UGU |
| 9705 | Nonsynonymous | UGU to UUU |
| 10846 | Nonsynonymous | AUG to AUU |
| 11042 | Nonsynonymous | GUU to UUU |
| 11083 | Nonsynonymous | UUG to UUU |
| 11417 | Nonsynonymous | GUU to UUU |
| 12208 | Nonsynonymous | AAG to AAU |
| 12464 | Nonsynonymous | GCA to UCA |
| 12467 | Nonsynonymous | GCC to UCC |
| 12491 | Nonsynonymous | GAC to UAC |
| 12572 | Nonsynonymous | GAU to UAU |
| 12578 | Nonsynonymous | GAU to UAU |
| 12582 | Nonsynonymous | AGU to AUU |
| 12773 | Nonsynonymous | GCU to UCU |
| 12793 | Nonsynonymous | AAG to AAU |
| 13513 | Nonsynonymous | CAG to CAU |
| 13571 | Nonsynonymous | GUU to UUU |
| 13771 | Nonsynonymous | UGG to UGU |
| 14044 | Nonsynonymous | UUG to UUU |
| 14118 | Nonsynonymous | CGC to CUC |
| 14229 | Nonsynonymous | AGC to AUC |
| 14718 | Nonsynonymous | AGG to AUG |
| 15193 | Nonsynonymous | GAG to GAU |
| 15418 | Synonymous | GUG to GUU |
| 15672 | Nonsynonymous | AGU to AUU |
| 15906 | Nonsynonymous | AGG to AUG |
| 16075 | Synonymous | CUG to CUU |
| 16377 | Nonsynonymous | CGU to CUU |
| 16396 | Nonsynonymous | CAG to CAU |
| 16858 | Nonsynonymous | AUG to AUU |
| 16897 | Nonsynonymous | AUG to AUU |
| 16912 | Nonsynonymous | UUG to UUU |
| 16957 | Nonsynonymous | UAG to UAU |
| 17278 | Nonsynonymous | AAG to AAU |
| 17302 | Nonsynonymous | AUG to AUU |
| 17338 | Nonsynonymous | CAG to CAU |
| 17427 | Nonsynonymous | UGU to UUU |
| 17679 | Nonsynonymous | CGC to CUC |
| 17808 | Nonsynonymous | AGA to AUA |
| 18149 | Nonsynonymous | GUU to UUU |
| 18281 | Nonsynonymous | GAC to UAC |
| 18325 | Nonsynonymous | AUG to AUU |
| 18756 | Nonsynonymous | CGU to CUU |
| 18762 | Nonsynonymous | UGA to UUA |
| 18898 | Nonsynonymous | UUG to UUU |
| 18973 | Nonsynonymous | AGG to AGU |
| 18984 | Nonsynonymous | UGG to UUG |
| 19542 | Nonsynonymous | UGA to UUA |
| 19645 | Nonsynonymous | AUG to AUU |
| 19656 | Nonsynonymous | AGG to AUG |
| 19684 | Nonsynonymous | AAG to AAU |
| 19999 | Nonsynonymous | GAG to GAU |
| 20002 | Nonsynonymous | UUG to UUU |
| 20134 | Synonymous | CCG to CCU |
| 20476 | Nonsynonymous | CAG to CAU |
| 20580 | Nonsynonymous | UGA to UUA |
| 21151 | Nonsynonymous | GAG to GAU |
| 21204 | Nonsynonymous | AGC to AUC |
| 21225 | Nonsynonymous | GGU to GUU |
| 21452 | Nonsynonymous | GUC to UUC |
| 21800 | Nonsynonymous | GAU to UAU |
| 21830 | Nonsynonymous | GUU to UUU |
| 21850 | Nonsynonymous | GAG to GAU |
| 22051 | Synonymous | GCG to GCU |
| 22335 | Nonsynonymous | UGG to UUG |
| 22436 | Nonsynonymous | GCA to UCA |
| 22468 | Synonymous | ACG to ACU |
| 22661 | Nonsynonymous | GUC to UUC |
| 22785 | Nonsynonymous | AGA to AUA |
| 23120 | Nonsynonymous | GCA to UCA |
| 23242 | Synonymous | CUG to CUU |
| 23405 | Nonsynonymous | GUU to UUU |
| 23593 | Nonsynonymous | CAG to CAU |
| 23755 | Nonsynonymous | AUG to AUU |
| 23856 | Nonsynonymous | CGU to CUU |
| 24095 | Nonsynonymous | GCU to UCU |
| 24348 | Nonsynonymous | AGU to AUU |
| 24368 | Nonsynonymous | GAC to UAC |
| 24755 | Nonsynonymous | GUG to UUG |
| 24794 | Nonsynonymous | GCU to UCU |
| 24928 | Synonymous | GUG to GUU |
| 25249 | Nonsynonymous | AUG to AUU |
| 25250 | Nonsynonymous | GUG to UUG |
| 25302 | Nonsynonymous | UGU to UUU |
| 25305 | Nonsynonymous | UGU to UUU |
| 25429 | Nonsynonymous | UGU to UUU |
| 25494 | Nonsynonymous | GAU to UAU |
| 25523 | Synonymous | CGG to CGU |
| 25534 | Nonsynonymous | UGU to UUU |
| 25563 | Nonsynonymous | GAG to UAG |
| 25606 | Nonsynonymous | AGC to AUC |
| 25644 | Nonsynonymous | GUU to UUU |
| 25677 | Nonsynonymous | GCU to UCU |
| 25691 | Nonsynonymous | UGG to UGU |
| 25740 | Nonsynonymous | GAG to UAG |
| 25767 | Nonsynonymous | GAG to UAG |
| 25770 | Nonsynonymous | GCU to UCU |
| 25775 | Nonsynonymous | UUG to UUU |
| 25785 | Nonsynonymous | GAA to UAA |
| 25979 | Nonsynonymous | UGG to UGU |
| 26063 | Nonsynonymous | UGG to UGU |
| 26109 | Nonsynonymous | GCC to UCC |
| 26144 | Synonymous | CGG to CGU |
| 26233 | Nongene | G to U |
| 26526 | Nonsynonymous | GCA to UCA |
| 26690 | Synonymous | CUG to CUU |
| 26730 | Nonsynonymous | GUU to UUU |
| 26775 | Nonsynonymous | GCU to UCU |
| 27147 | Nonsynonymous | GAC to UAC |
| 27225 | Nonsynonymous | CAG to CAU |
| 27226 | Nonsynonymous | GUU to UUU |
| 27327 | Nonsynonymous | AAG to AAU |
| 27382 | Nonsynonymous | GAU to UAU |
| 27877 | Nonsynonymous | UGU to UUU |
| 28001 | Synonymous | CCG to CCU |
| 28077 | Nonsynonymous | GUG to UUG |
| 28086 | Nonsynonymous | GCU to UCU |
| 28089 | Nonsynonymous | GGU to UGU |
| 28191 | Nonsynonymous | GUG to UUG |
| 28202 | Synonymous | UCG to UCU |
| 28280 | Nonsynonymous | GAU to UAU |
| 28300 | Nonsynonymous | CAG to CAU |
| 28378 | Synonymous | GCG to GCU |
| 28392 | Nonsynonymous | CGU to CUU |
| 28541 | Nonsynonymous | GCU to UCU |
| 28655 | Nonsynonymous | GAC to UAC |
| 28703 | Nonsynonymous | GAU to UAU |
| 28727 | Nonsynonymous | GCU to UCU |
| 28739 | Nonsynonymous | GCA to UCA |
| 28812 | Nonsynonymous | AGU to AUU |
| 28827 | Nonsynonymous | CGU to CUU |
| 28842 | Nonsynonymous | AGU to AUU |
| 28851 | Nonsynonymous | AGU to AUU |
| 28985 | Nonsynonymous | GGC to UGC |
| 29027 | Nonsynonymous | GCU to UCU |
| 29140 | Nonsynonymous | CAG to CAU |
| 29254 | Synonymous | UCG to UCU |
| 29431 | Nonsynonymous | CAG to CAU |
| 29462 | Nonsynonymous | GCU to UCU |
| 29465 | Nonsynonymous | GCA to UCA |
| 29511 | Nonsynonymous | AGU to AUU |
| 29543 | Nongene | G to U |
| 29557 | Nongene | G to U |
| 29628 | Nonsynonymous | CGU to CUU |
| 29688 | Nongene | G to U |
| 29692 | Nongene | G to U |
| 29711 | Nongene | G to U |
| 29736 | Nongene | G to U |
| 29742 | Nongene | G to U |
| 29747 | Nongene | G to U |
| 29764 | Nongene | G to U |
| 29781 | Nongene | G to U |
| 29864 | Nongene | G to U |

Figure S2. Sequence frequency logo based on nucleotide frequencies surrounding G to U mutation sites in SARS-CoV-2 genomes. We see that there is no specific context for these mutations.


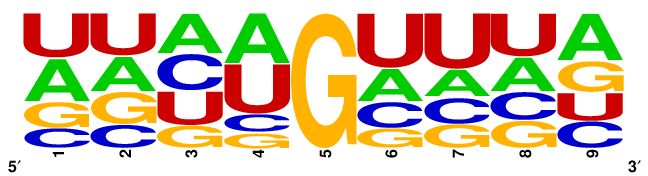

Supplement: Supplemental Information 1 [file peerj-08-9648-s001.docx]
